# Qualitative study on experiences reported by people with complex wounds In Primary Health Care

**DOI:** 10.1590/0034-7167-2024-0628

**Published:** 2026-02-23

**Authors:** Dalila Reis Barbosa, Caio Vieira de Barros Arato, Rodrigo Almeida Bastos, Norma Sueli Gonçalves Reche, Michelli Caroliny de Oliveira, Egberto Ribeiro Turato, Luciane Miranda Guerra

**Affiliations:** I Universidade Estadual de Campinas. Campinas, São Paulo, Brazil.

**Keywords:** Ulcer, Primary Health Care, Qualitative Research, Public Health, Hermeneutics

## Abstract

**Objectives:**

to explore expressed and unexpressed senses and meanings that may reveal subjective elements necessary for patients’ emotional management.

**Methods:**

a clinical-qualitative approach was used within the Brazilian Health System with individuals who have had chronic wounds for more than six months. Data were collected through in-depth individual interviews, audio-recorded and transcribed verbatim using a semi-structured script. The sample was calculated based on theoretical saturation. The corpus was analyzed using the “Seven Steps” technique, and the theoretical framework supporting the analysis and discussion was health psychology.

**Results:**

eight interviews were conducted with patients treated at Family Health Units. The meanings and significances characterized three categories: distress beyond physical pain; communication between patient and professional; and religiosity as a coping strategy.

**Final Considerations:**

living with difficult-to-heal wounds compromises adherence to treatment and self-care.

## INTRODUCTION

People living with complex wounds often experience severe physical and psychosocial changes such as pain, impaired mobility, poor self-care, inability to perform activities of daily living, anxiety, depression, shame, changes in body image, and emotional problems. Furthermore, they often experience discrimination and consequent social isolation^(1,2)^.

A wound that persists for several years can leave an individual feeling apathetic, unmotivated, and complacent. The daily routine of treatments and dressings with no end in sight can lead to complacency and a lack of faith in the possibility of a cure. This situation is generally perceived as hopeless, and its treatment is perceived without enthusiasm, performed as a routine obligation^(3–4)^.

Wounds whose healing process lasts more than three months are called complex wounds^(5–6)^ and, in this case, are generally associated with infectious processes and systemic pathologies. This condition affects millions of people worldwide, and approximately 5% of the adult population in Western countries suffers from this condition^(7–8)^.

Given that the set of biopsychosocial changes caused by complex wounds generally weakens the mental health of those affected^(7)^, providing healthcare to these individuals is a major challenge, not only for those experiencing the wound but also for those providing care. Therefore, the interaction between patient and healthcare professional can be challenging, revealing anguish, frustration, anxiety, and other emotions on both sides. Qualitative research addresses the subjectivity of the individuals studied and seeks to understand the relationships, representations, and opinions as a product of the interpretations present in the social interactions that the investigated agents have, revealing how they live, feel, and think about their life paths^(9–10)^.

Although numerous studies address the physical and psychosocial repercussions of complex wounds, the literature predominantly focuses on quantitative research focused on analyzing patients’ quality of life, relegating the understanding of the subjectivities involved to the background. Few qualitative studies attempt to deeply understand the discourse of individuals living with this condition, capturing the nuances of their feelings, meanings, and experiences. This gap is especially relevant, since the interaction between patients and healthcare professionals often reveals anxieties, frustrations, and other emotions that impact the clinical and emotional management of these individuals. The lack of studies that prioritize the symbolic dimension of the experiences of people living with complex wounds represents a barrier to the development of comprehensive and humanized care that transcends technical procedures and includes subjectivity as a central element. This study should, therefore, serve as a subsidy so that healthcare professionals, knowledgeable about the symbolic content of patients’ relationship with their wound, can assist a person living with a complex wound beyond the technical procedure, i.e., considering each one’s subjectivity.

## OBJECTIVES

To explore the manifested and unmanifested senses and meanings, which can reveal subjective elements necessary for patients’ emotional management.

## METHODS

### Ethical aspects

The study was conducted in accordance with national and international ethics guidelines, and was approved by the *Faculdade de Odontologia de Piracicaba* Research Ethics Committee, whose opinion is attached to this submission.

Written Informed Consent Form (ICF) was obtained from all individuals involved in the study.

### Theoretical-methodological framework

The interviews were analyzed within the health psychology framework and used psychodynamics to capture and interpret manifest or latent content. Therefore, it was a framework that contained concepts drawn from the body of knowledge in human sciences for use in clinical-qualitative research. Some of the psychodynamic concepts of interest in interpreting these data were cited: ego defense mechanisms, as well as failed acts and lapses, as expressions not explicitly intended, but manifested and charged with meanings capable of being known and revealing inner truths^(11)^.

### Study design

This clinical-qualitative study, with a convenience sample, was developed for specific use in clinical care settings, conducted from July 2023 to September 2024. The COnsolidated criteria for REporting Qualitative research was adopted to guide the study methodology.

The clinical-qualitative methodology seeks to deeply understand the complexities of human experience. The main strength of this method lies in its validity—the ability to construct data collection instruments that are coherent and relevant. This is due to the fact that, in qualitative research, the logic of replicability is not always applicable, since subjects carry multiple meanings and interpretations. Another relevant aspect is its flexibility, which allows its application in various areas of scientific research in health.

### Methodological procedures

This study was conducted by the lead author, a trained researcher acculturated in the research environment (primary care), and a nurse with 28 years of experience at the research site. She specializes in dermatological nursing. The researcher does not work at the health units where the research was conducted, but rather at another Family Health Unit (FHU). The choice of a health unit different from her own was intentional to avoid measurement bias due to supposed proximity between her and the interviewees. Furthermore, the environment, habits, and work organization are very similar to those of her FHU, which led to her acculturation to the research site. The researcher assumed that subjective elements observed in patients living with complex wounds have a significant impact on the therapeutic process of this condition.

Two training and acculturation interviews were conducted, transcribed, and subsequently discussed among pairs of researchers from the Qualitative Research and Study Group (GEPEQ). They analyzed them and made appropriate adjustments to their format and content, training and enhancing the researcher’s data collection skills. These training interviews were not included in the results.

The inclusion criterion for interviewees was being a patient between the ages of 18 and 80, living with complex wounds of vascular and/or neurogenic etiology in the lower limbs, with a duration of more than six months, receiving care within the Primary Health Care (PHC) system in the municipality of Amparo, São Paulo, and receiving care at one of the following FHUs: Silvestre, Camanducaia, Moreirinha, Santa Maria, and Arcadas. The exclusion criterion was having speech or cognitive impairments that prevented the understanding of their responses.

### Study setting

The five FHUs in the municipality of Amparo, São Paulo, that had the highest number of users monitored were selected, namely FHU Silvestre, FHU Camanducaia, FHU Moreirinha, FHU Santa Maria and FHU Arcadas.

After the study project received ethical approval from the Research Ethics Committee, the principal researcher contacted the healthcare facility, which promptly provided the names and contact details of patients who met the inclusion criteria. Interviews were then scheduled in advance by phone or WhatsApp®, during which the researcher briefly explained the objectives and the interview process. On the scheduled day, the interviewer read the ICF to the interviewees, detailing the purpose and the interview. She asked if they had any questions and clarified what was necessary. The interviews began only after each participant signed the ICF. The interviews took place from September 2023 to April 2024, and all were conducted in a private, private setting, either at the facility of origin or at the interviewee’s home (as chosen by the interviewee), ensuring their privacy and ensuring the confidentiality of the information collected. Only the interviewer and the interviewee participated.

### Data source

This is a convenience-based study. The target audience was adults living with complex wounds in a medium-sized municipality in the countryside of São Paulo state.

### Data collection and organization

Data collection was conducted through semi-directed, in-depth individual interviews, which were audio-recorded and transcribed verbatim using a semi-structured script. The interviews lasted approximately 20 to 40 minutes, and each participant was interviewed only once. Participant data was protected by code identification. The verbatim transcripts will not be released to maintain the privacy and reliability of the information collected.

The triggering question for the interview was:

– What is it like for you to have a complex wound?

During the interview, additional questions were asked, based on a semi-structured script, with the following questions:

– How long have you had the wound?
– Have you always been cared for at FHU?
– How do your family/friends behave towards you and your wound?
– What is the work situation like for you? Are you able to work?
– And what about social life? How is it?

The criterion of theoretical data saturation was adopted, discussed for each category by GEPEQ research peers, to determine the number of individuals interviewed.

All data from this research are deposited in the Open Science Framework (OSF), available through the link https://osf.io/5gxad and DOI 10.17605/OSF.IO/5GXAD. The subjects were identified using codes in order to maintain the confidentiality of their identity.

### Data analysis

The data were analyzed using the clinical-qualitative content analysis technique, by peer researchers in research meetings of the GEPEQ group, following the seven steps^(12)^:

1. Editing material – all transcribed interviews;
2. Text skimming – the researcher makes a free reading of the reported life experiences;
3. Construction of units of analysis – researchers identify meanings, select speech fragments and develop initial reflections on each fragment;
4. Construction of meaning codes – grouping similar units of analysis, thus structuring the first meaning codes;
5. Category construction – organization of material for analysis by all participants with a view to grouping the meaning codes;
6. Discussion – a dialogue with the available literature;
7. Validity – critical reflection on the processes carried out at each stage.

## RESULTS

Eight users were included and interviewed. There were no dropouts. Their ages ranged from 50 to 80 years (average 65 years), with two males and six females. Three users had previously undergone amputation; four live with diabetes (two of whom had previously undergone limb amputations); and the others have other vascular pathologies. The duration of the wounds ranged from one to 50 years, with three people having wounds lasting one year, and the others lasting more than ten years. Among the interviewees, three had a history of limb amputation: two toes and one both legs. Only one person worked as a general services assistant. Regarding their place of residence, four interviewees had to leave their homes temporarily or permanently to live with someone else due to total or partial dependence for self-care. Two interviewees demonstrated greater fragility and emotional difficulty dealing with the wound and other personal issues. In these cases, the interview was interrupted, and the interviewees were referred for psychological assessment in the municipal network.

Three categories were consolidated, which are described and illustrated below with reports of the senses, meanings and perceptions of people living with the condition in relation to the persistence of their wounds:

1. No longer has a social life: distress beyond physical pain;
2. Patient-professional communication;
3. Religiosity as a coping strategy.

### 1) No more social life: distress beyond physical pain

In addition to the present and permanent physical pain that accompanies the interviewees, there is the worsening of perceived distress, which manifested itself subjectively through feelings of various losses (of autonomy, of social life, of the role in the family, etc.), by frustrations and by stigma.

[…] *it’s just that we want to go out, get back to our normal lives, right?!* (P2)

[…] *my life, right, there’s no social life anymore! […] I think all this affects me, yes. […] there’s nowhere to go without shoes, right? I haven’t gone out in a long time […] we feel a little useless, right?* (P4)

*I feel really bad. It gives you a bad feeling, you don’t feel comfortable. You look ugly. Oh, it’s like something in you is going away! You want to hide it! It’s a mark that really leaves its mark! So, you keep hiding it […].* (P8)

The lack of prospects permeates people’s routines and brings discouragement and pessimism.

[…] *I feel sad, discouraged, it hurts, it burns, itches, it itches. It hurts day and night, taking medicine doesn’t help, so I get discouraged […] taking medicine and everything and I don’t see any results.* (P7)

[…] *I was […] I don’t know, distressed, discouraged, because it hurt so much, right? It was a deep sock. And it burned my leg so badly. It was on fire. And I looked at it like that, I did everything, and it didn’t get better.* (P1)

*I suffered a lot, a lot of pain! […] when I go to the health center [FHU], to get my foot bandaged, I get nervous. I get nervous, thinking the worst. I can’t handle another surgery; it’s going to be difficult. That’s my fear.* (P5)

There are distortions between what is said and what is understood, and the impact this has on the emotionally involved person can stay with them for the entire period they remain with the wound. From this emerged a category related to communication:

### 2) Patient-professional communication

*Then, before amputating the second toe, the doctor explained to me, “You’re at huge risk of losing this foot”. Oh my God, I was even more distressed, wasn’t I!* (P2)

[…] *there was a doctor who said to me, “What was the point of keeping your foot if it wasn’t of any use to you?”. Because she said, “If you have your foot, the doctor didn’t amputate your foot, but you won’t be able to use it”.* (P4)

*And the doctor told me it’s due to the blood, right, circulation.* (P3)

There seems to be a dissonance between speaking and listening. What is said often differs from what is heard and interpreted. The belief that the wound is incurable can be reinforced by communication, as in the statements below from P7 and P6:

*The doctor himself said it’s chronic, there’s no cure. It gets better, then it comes back […] chronic because it’s been 50 years […].* (P7)

*[The doctor said] “Oh, you think there’s going to be a cure? There’s no cure, no […]”.* (P6)

This lack of prospects for healing opens the door to frustration, fantasies, and a belief in the intangible. This is reflected in the conversations about religion, from which a category related to religiosity emerged:

### 3) Religiosity as a coping strategy

Religion and faith are always present in people’s daily lives, and this can negatively or positively impact the lives of individuals undergoing treatment for complex wounds. Faith, in these cases, has two aspects: faith that sustains optimism and the hope for healing; and faith that tends toward negativity, which makes the person a victim of punishment.

*I don’t know if you believe it or not […] I think they did something to me.* (P1)

*Look, I’m an evangelical. I have a lot of faith in God […]. I put it in God’s hands, and I hope he blesses me.* (P5)

*So, I put it in God’s hands. God knows what he’s doing. So, sooner or later, it will heal. I have faith.* (P1)

*But I was more like this […] with that faith that I’m healing.* (P2)

*[…] the one who healed me was God. God is everything, right?! So, by God, I might be cured, but by me, no.* (P6)

There are current superstitions among patients with complex wounds that these injuries can be “punishments”, “blood stains”, that there is no treatment or cure, or even about the imminence of death due to scarring, as in the case of P1, who has had the wound for more than 30 years and tells the following story, revealing fear of the proximity of death.

*I didn’t believe in anything anymore!! […] I don’t know if you believe in faith healers, that kind of thing […] that a woman went and blessed [herself], and the wound closed, but it didn’t last long. The wound closed, but the person didn’t last long.* (P1)

*I don’t know if you believe it or not, but things like that, poorly done, I believe in those things […] I think they did something to me.* (P1)

## DISCUSSION

The reports revealed a lack of communication between professionals and patients. Due to the pathology of the wounds, the individual must change their patterns and lifestyle, and begins to live according to their problem, giving up the things they loved most. When reflecting on the need for changes in routine and daily life, interviewees’ statements reveal an important clinical fact that explains one of the difficulties in wound treatment: treatment adherence.

During the interviews, it was clear that faith brings ambiguous consequences, both optimistic and pessimistic, and these feelings are consistent with the progression of these individuals’ conditions.

Understanding the subjective dimension of distress for a patient living with a complex wound requires going beyond the physical, technical, and technological dimensions of care for this condition. Therefore, this means exploring elements that go beyond the biomedical paradigm, which structures healthcare training.

The social and subjective dimensions of healthcare are inseparable, and this is a fact. After all, disease “inhabits” a body, which is embedded in a society and communicates something to us through its pathology, its illness, and its social reality. Therefore, simply medicalizing is insufficient. We must examine what disease and health represent, what senses and meanings are experienced and faced, what they say about lived space, and about social and individual dynamics^(13,14)^.

Research into the experiences of patients with complex wounds revealed the pain that stems from subjectivity, from what is said between the lines of conversation. Pain, considered the fifth vital sign, is not only physical but persists and manifests itself in different ways, exposing the imposed limitations, the acquired dependence, and, subtly, the expectation and hopelessness that accompany the patient. Thus, the first thematic category of this study emerged:

### No more social life: distress beyond physical pain

Living with a patient with a complex wound and perceiving their physical and psychological distress makes us reflect that this condition brings a series of changes to the lives of not only the people with a wound, but also their families, who are often not prepared to understand all the aspects involved in this problem^(3)^.

Living with a scar that requires routine care can be distressing and often desperate^(3)^. The physical scar the wound leaves behind represents a problem that is not only perceptible to the individual but also to everyone around them, limiting interpersonal relationships^(15,16)^. A person’s self-perception is not only related to perception and senses, but also involves mental representations and figurations of themselves and others, and the contact with actions and emotions arising from these experiences. Individuals with a complex wound carry the burden of a visible disease that is evident on their skin and a sadness that weighs on their soul, causing psychological distress and a loss of quality of life^(3)^. Therefore, it is a subjective pain.

Studies on the perceptions of patients with complex wounds indicate that the most comprehensive observation is the burden that patients suffer from their wounds and how this leads them to become passive observers of their care^(17)^.

In the daily lives of people with wounds, there is distress due to doubts and anxieties regarding treatment, as well as anxiety about seeing the wound progress toward improvement. It is clear that the wound may not be just a physical injury, but something that hurts without necessarily requiring sensory stimulation, a mark, an irreparable loss—in other words, something beyond an incurable disease^(18)^. In this sense, the results of a study conducted with patients distress from lower limb injuries corroborate the findings of this study, as participants report that concern arises from uncertainty about recovery and fear of complications, leading to constant worry. Similarly, frustration stems from physical limitations and the slow healing process, which generates dissatisfaction and exacerbates emotional distress^(18)^.

Healthcare for these patients is typically an opportunity to access care that can alleviate these anxieties. However, what we observed was that significant dissonances can occur in this patient-professional encounter, which cannot only deepen existing anxieties but also create new complications. This perception of the patients interviewed gave rise to a category related to patient-professional communication.

### Patient-professional communication

The way in which bad news is communicated directly influences the next stages of treatment, i.e., the user’s follow-up of treatment is strongly defined based on the effectiveness of communication^(19)^. In addition to this information and how it is interpreted, it is necessary to consider the baggage that weighs on the person with a complex wound, which can leave deep scars that accompany a person for a long period, complicating the situation, as well as contributing to the negative feelings that permeate the daily lives of these people^(20–21)^.

The way professionals express the complexity of the case can lead patients to not believe in the therapeutic processes necessary for their problem^(20)^.

Healthcare professionals should become more familiar with soft and relational technologies, since the act of caring is intrinsically linked to communication, which is a soft technology. More than simply providing accurate information, therapeutic communication must consider the patient’s distress, prognosis, and ability to understand, perceived through qualified listening and subjective cues. Wound patients, already fearful of chronicity, can often distort the information they receive, and this information can compound beliefs about the incurable wound.

Patients’ subjective needs require professionals to adopt psychodynamic approaches, fostering understanding and acknowledging these needs. These approaches are also subjective and are as important as the clinical conduct protocol itself^(21)^.

In the absence of these professional attitudes, what can be identified is that patients’ distress gives rise to superstitions that can potentially lead them to abandon treatment, but, on the other hand, generate the need for belief in the supernatural to seek hope and healing. Thus, religiosity was an emerging category in the statements, demonstrating its importance in the therapeutic process.

### Religiosity as a coping strategy

Religious belief is an important part of culture, the principles and values people use to shape their judgments and information processing. Spirituality brings hope and perspectives for a better future, for a better quality of life, and for the hope of healing^(22,23)^.

Faith and religiosity are recurring themes in studies exploring people’s perceptions of the meaning of complex wounds in their lives. Corroborating the findings of this research, an article found that spirituality was exalted as something sacred^(24)^. Patients defined God as the being above all, and faith was identified as a path to well-being, transcending the soul and giving them hope for healing and scarring of the complex injury. However, there are superstitions regarding complex wounds that work in the opposite way, bringing hopelessness, since they present the wound as a “moral reckoning” between God and individuals.

While respecting the practical importance of spirituality, religiosity, and faith from a therapeutic perspective, if we want to delve deeper into the meanings these patients attribute to their illness, we need to understand this phenomenon from a psychodynamic perspective. It is common among people experiencing great challenges, distress, and anguish to use ego defense mechanisms such as regression, which functions like a child’s trust in the protection of their parents. It is a way of protecting oneself from the distress and negativity that permeate complex cases. Regression is a defense mechanism characterized by an individual’s return to previous forms of thought development and style of relating to their environment. It is characterized by a decreased ability to endure frustration, dependence on social groups, and, as in the cases of interviewees, magical thinking, in which patients believe in and surrender to the omnipotence of the divine^(25–26)^.

When the healthcare team has basic psychodynamic knowledge of these phenomena and their impact on treatment, they can better support the individual in the most challenging moments, such as when expectations are frustrated or when new deprivations and limitations are imposed. It will be frustrating for a patient who strives for faith to recognize the frustration of the magical elements that once guided them. At this point, the team needs to be attentive to welcoming and seeking to rekindle hope in the patient. The psychotherapeutic approach denotes having an attitude of acceptance of the patient’s anxieties. It is important to emphasize that the psychotherapeutic approach of healthcare professionals is not to provide therapy, but to have an attitude that welcomes patients’ anxieties. It promotes a non-protocolized clinical, therapeutic, and interpersonal relationship, essential for subsequent technical therapeutic follow-up^(22)^.

On the other hand, by speaking freely about the meanings and significance of the wound in their lives, these patients were able to process their distress, even if only momentarily, which, certainly, in itself, is already a therapeutic moment.

### Study limitations

The interviews in this study, because they were conducted by a researcher who is a nurse, in the same healthcare network where the interviewees are treated, may carry a certain response bias from these individuals, who may have omitted some question due to embarrassment. In this sense, care was taken to ensure that the interviewer did not interview patients she had treated, in order to mitigate this potential bias. In addition to this methodological precaution, it is also necessary to consider that while there is a risk of bias, the researcher’s experience in the field also allows her to delve deeper into data collection, due to her familiarity with the topic and the natural course of this condition. Therefore, it is important to note that, despite the richness of the results obtained, there may still be more elements to be explored, for which reason it is suggested that this research be continued with an interviewer not associated with the service.

### Contributions to health, nursing or public policy

The results of this study can provide healthcare professionals with subjective insights gathered from the experiences of people living with complex wounds, which can guide care for these patients in a way that is more consistent with their needs. This uniqueness, in addition to making the patient-professional encounter more welcoming, will allow healthcare professionals to transform merely biological care (focused on the wound) into an act that considers the integrity of these individuals. Attention to the subjectivity of people with wounds, therefore, is the path to recognizing their distress and respecting their numerous losses and limitations.

Specifically, regarding the training of PHC professionals, this study contributes to pointing the way toward patient-centered care. It is known that, despite numerous technological and knowledge advances in wound care, some factors pose challenges and contribute to worsening, such as tobacco use, dietary restrictions, signs of infection, odor characteristics, and pain assessment^(27)^. Building and strengthening the patient-professional bond appears crucial, as it is central to user adherence to the service and professional recommendations. However, it must be considered that the difficulties and changes in the daily lives of people living with a complex wound for a prolonged period, as well as the repercussions for the individual and family, do not facilitate this adherence; quite the opposite. The hopelessness that permeates the days, the procedures with no end in sight, and the frustration with care without progress are part of these people’s daily lives for months and years.

When healthcare professionals are aware of this, they certainly empathize, include in their treatment plans means to overcome practical difficulties that go beyond merely technical care, and welcome these patients differently, focusing their care on each patient individually. The reports in this study contribute, therefore, by highlighting some of the meanings that wounds hold for those who carry them and, in this way, prepare PHC professionals to face them. From an organizational perspective, PHC units, recognizing the challenging meanings reported in this study that living with wounds brings to those who carry them, can organize themselves to receive these patients quickly and with the greatest possible privacy within the unit’s facilities, avoiding long waits in shared rooms. Furthermore, they can conduct home visits with appropriate frequency for each case and, above all, promote spaces for discussion about patient-centered care for their staff.

## FINAL CONSIDERATIONS

Complex wounds, persistent for a prolonged period, not only leave marks on the skin, but also provoke a sadness that weighs heavily on the soul, bringing psychological distress resulting from the losses and the adaptations needed to adapt to their reality.

Knowledge of the meanings these wounds hold for the patients affected by them is essential for the professionals involved to adopt a psychotherapeutic approach in these treatments, which tends to increase the resolution of cases, since their management goes beyond pathophysiological knowledge.

The distress experienced by people with complex wounds must be recognized by the caregivers of these patients, in order to quality the therapeutic relationship to the extent that, by considering them, the professional can bring their recommendations closer to the patient’s reality, empathize with their experiences and, furthermore, improve patient-professional communication, giving their discourse coherence with the subjectivity of each case.

## Data Availability

The research data are available in a repository: https://doi.org/10.17605/OSF.IO/5GXAD.
