# Supplementary material for: Qualitative study on experiences reported by people with complex wounds In Primary Health Care
Source: Rev Bras Enferm. 2026 Feb 23;79:e20240628. doi: 10.1590/0034-7167-2024-0628 (PMC12933937; doi:10.1590/0034-7167-2024-0628)
Supplement: Translation

# Translation: Estudo qualitativo sobre experiências relatadas por pessoas com feridas complexas na Atenção Primária à Saúde

**DOI:** 10.1590/0034-7167-2024-0628pt

**Authors:** Dalila Reis Barbosa, Caio Vieira de Barros Arato, Rodrigo Almeida Bastos, Norma Sueli Gonçalves Reche, Michelli Caroliny de Oliveira, Egberto Ribeiro Turato, Luciane Miranda Guerra

**Affiliations:** 1 Universidade Estadual de Campinas. Campinas, São Paulo, Brasil.

**Keywords:** Úlcera, Atenção Primária à Saúde, Pesquisa Qualitativa, Sistema Único de Saúde, Ferimentos e Lesões

## Abstract

**Objetivos:**

explorar sentidos e significados manifestados e não manifestados que podem revelar elementos subjetivos necessários ao manejo emocional dos pacientes.

**Métodos:**

abordagem clínico-qualitativa, realizado no âmbito do Sistema Único de Saúde com pessoas que possuem feridas crônicas há mais de seis meses. Dados foram coletados através de entrevistas individuais em profundidade, audiogravadas e transcritas na íntegra com auxílio de roteiro semiestruturado. Amostra foi composta por saturação teórica. O *corpus* foi analisado através da técnica “*Seven Steps*”, e o referencial teórico de suporte da análise e discussão foi o da psicologia da saúde.

**Resultados:**

foram realizadas oito entrevistas com usuários acompanhados nas Unidades de Saúde da Família. Os sentidos e significados caracterizaram três categorias: sofrimento para além da dor física; comunicação entre paciente e profissional; e religiosidade como estratégia de enfrentamento.

**Considerações Finais:**

a convivência com as feridas de difícil cicatrização compromete a adesão ao tratamento e autocuidado.

## INTRODUÇÃO

As pessoas que vivem com feridas complexas apresentam frequentemente alterações físicas e psicossociais intensas, como dor, prejuízos na mobilidade, déficit no autocuidado, incapacidade de realizar as atividades de vida diária, ansiedade, depressão, vergonha, alterações na imagem corporal e problemas emocionais. Além disso, geralmente sofrem discriminação com consequente isolamento social^(1,2)^.

A ferida que permanece por vários anos pode colocar o indivíduo em uma situação de apatia, desmotivação e comodismo. A rotina diária de tratamento e curativos sem previsão de término pode trazer acomodação, além de descrédito na possibilidade de cura. Em geral, essa situação é percebida como algo sem solução, e seu tratamento é compreendido sem anseios, executado como uma obrigação rotineira^(3,4)^.

A ferida, cujo processo cicatricial se prolonga por mais de três meses, é denominada de complexa^(5,6)^ e, nesse caso, geralmente está associada a processos infecciosos e patologias sistêmicas. Esta condição acomete milhões de pessoas em todo o mundo, e aproximadamente 5% da população adulta no território ocidental sofre de tal agravo(^(7,8)^).

Tendo em vista que o conjunto das alterações biopsicossociais causadas pela ferida complexa em geral debilita a saúde mental das pessoas acometidas^(1)^, o cuidado em saúde dessas pessoas representa um grande desafio a ser enfrentado, não só por quem vivencia, mas também por quem cuida. Em face disso, a interação entre o paciente e o profissional de saúde pode ser desafiadora, ao revelar angústias, frustrações, ansiedades e outras emoções de ambas as partes. Pesquisas de abordagem qualitativa abordam o conhecimento da subjetividade dos indivíduos investigados, buscando apreender as relações, representações e opiniões como produto das interpretações presentes nas interações sociais que os agentes investigados possuem, desvelando como vivem, sentem e pensam seus percursos de vida^(9,10)^.

Apesar de inúmeros estudos abordarem as repercussões físicas e psicossociais das feridas complexas, a literatura apresenta uma predominância de pesquisas quantitativas voltadas à análise da qualidade de vida dos pacientes, relegando a segundo plano o entendimento das subjetividades envolvidas. Poucas são as investigações qualitativas que se propõem a compreender, de maneira aprofundada, o discurso dos indivíduos que convivem com essa condição, capturando as nuances de seus sentidos, significados e experiências. Tal lacuna é especialmente relevante, uma vez que o processo de interação entre os pacientes e profissionais de saúde muitas vezes desvela angústias, frustrações e outras emoções que impactam o manejo clínico e emocional dessas pessoas. A ausência de estudos que privilegiem a dimensão simbólica das vivências das pessoas que convivem com feridas complexas representa uma barreira para construção de um cuidado integral e humanizado que transcenda o procedimento técnico, incluindo a subjetividade como elemento central na assistência. Esse estudo deve, portanto, servir como subsídio para que os profissionais de saúde, conhecedores do simbólico contido na relação do paciente com sua ferida, possam assistir a pessoa que convive com ferida complexa para além do procedimento técnico, ou seja, considerando a subjetividade de cada um.

## OBJETIVOS

Explorar os sentidos e significados manifestados e não manifestados, que podem revelar elementos subjetivos necessários ao manejo emocional dos pacientes.

## MÉTODOS

### Aspectos éticos

O estudo foi conduzido de acordo com as diretrizes de ética nacionais e internacionais, e foi aprovado pelo Comitê de Ética em Pesquisa da Faculdade de Odontologia de Piracicaba, cujo parecer está anexado à presente submissão.

O Termo de Consentimento Livre e Esclarecido (TCLE) foi obtido de todos os indivíduos envolvidos no estudo por meio escrito.

### Referencial teórico-metodológico

A análise das entrevistas foi feita segundo o referencial da psicologia da saúde, utilizando a psicodinâmica para captar e interpretar conteúdos manifestos ou latentes. Foi, portanto, um referencial que continha conceitos trazidos do corpo de conhecimentos de áreas das ciências humanas para uso na pesquisa clínico-qualitativa. Citaram-se algumas das concepções psicodinâmicas de interesse na interpretação desses dados, como mecanismo de defesa do ego, bem como atos falhos e lapsos, enquanto expressões não almejadas explicitamente, porém manifestadas e carregadas de significados passíveis de serem conhecidos e reveladores de verdades interiores^(11)^.

### Tipo de estudo

Estudo clínico-qualitativo, com amostra de conveniência, desenvolvido na particularização e refinamento para uso específico no enquadre clínico-assistencial, realizado de julho de 2023 a setembro de 2024. O instrumento *Consolidated criteria for REporting Qualitative research (Coreq)* foi adotado para nortear a metodologia do estudo.

A metodologia clínico-qualitativa busca compreender, em profundidade, as complexidades da experiência humana. A principal força desse método reside em sua validade, ou seja, na capacidade de construir instrumentos de coleta de dados que sejam coerentes e pertinentes. Isso se deve ao fato de que, nas pesquisas qualitativas, a lógica da replicabilidade nem sempre é aplicável, uma vez que os sujeitos são portadores de múltiplos significados e interpretações. Outro aspecto relevante é a flexibilidade, que permite sua aplicação em diversas áreas de investigação científica em saúde.

### Procedimentos metodológicos

O presente estudo foi realizado pela autora principal, pesquisadora treinada e aculturada no ambiente de pesquisa (atenção primária), enfermeira, com 28 anos de trabalho no local de desenvolvimento da pesquisa. É especialista em enfermagem dermatológica. A pesquisadora não atua nas unidades de saúde onde a pesquisa foi realizada, mas sim em outra Unidade de Saúde da Família (USF), e a escolha de unidade de saúde diferente da sua de atuação foi proposital, em função de se evitar viés de aferição por suposta proximidade entre a mesma e os entrevistados. Por outro lado, tratam-se de ambiente, hábitos e organização de trabalho muito semelhantes aos de sua USF de atuação, o que ensejou a sua aculturação ao local de pesquisa. A pesquisadora partiu do pressuposto de que elementos subjetivos observados em pacientes que convivem com feridas complexas têm importante interferência no processo terapêutico desse agravo.

Foram realizadas duas entrevistas de treinamento e aculturação, as quais foram transcritas e posteriormente discutidas entre os pares de pesquisadores do Grupo de Estudos e Pesquisas Qualitativas (GEPEQ), que as analisaram e fizeram os devidos ajustes de forma e conteúdo, treinando e aprimorando a capacidade de coleta dos dados por parte da pesquisadora. Essas entrevistas de treinamento não foram incluídas no *corpus* dos resultados.

O critério de inclusão dos entrevistados foi ser paciente na faixa etária de 18 a 80 anos, que convive com feridas complexas, de etiologia vascular e/ou neurogênica em membros inferiores, com evolução superior a seis meses, assistido no âmbito da Atenção Primária à Saúde (APS) do município de Amparo, São Paulo, e seja atendido por uma das seguintes USF: Silvestre, Camanducaia, Moreirinha, Santa Maria e Arcadas. O critério de exclusão foi o paciente possuir impossibilidades na fala ou cognição que inviabilizaram a compreensão de suas respostas.

### Cenário do estudo

Foram selecionadas as cinco USFs do município de Amparo, São Paulo, que apresentavam maior número de usuários acompanhados, ou seja, USF Silvestre, USF Camanducaia, USF Moreirinha, USF Santa Maria e USF Arcadas.

Após aprovação ética do projeto deste estudo pelo Comitê de Ética em Pesquisa, a pesquisadora principal fez contato com a unidade de saúde, que prontamente cedeu os nomes e contatos dos pacientes que preenchiam os critérios de inclusão. As entrevistas foram, então, previamente agendadas por telefone ou *WhatsApp*, quando então a pesquisadora explicou brevemente seus objetivos e como seria a entrevista. No dia marcado, a entrevistadora leu o TCLE aos entrevistados, detalhando melhor o objetivo e a entrevista, perguntou se tinham alguma dúvida, esclareceu o que foi necessário e, somente após a assinatura de cada um ao TCLE, as entrevistas se iniciaram. As entrevistas ocorreram de setembro de 2023 a abril de 2024, e todas foram realizadas em ambiente privativo, reservado, na unidade de origem ou no domicílio (conforme a escolha do entrevistado), visando sua privacidade e confiabilidade das informações colhidas. Somente entrevistadora e entrevistado participaram.

### Fonte de dados

Trata-se de estudo com seleção de sujeitos por conveniência. O público-alvo foram pessoas adultas que convivem com feridas complexas de um município de médio porte do interior do estado de São Paulo.

### Coleta e organização dos dados

A coleta de dados foi feita por meio de entrevistas individuais semidirigidas, em profundidade, audiogravadas e transcritas na íntegra com auxílio de roteiro semiestruturado. As entrevistas duraram cerca de 20 a 40 minutos, e cada participante foi entrevistado uma única vez. Os dados dos participantes foram protegidos através da identificação por códigos. As falas, transcritas na íntegra, não foram divulgadas, de modo a manter a privacidade e a confiabilidade das informações colhidas.

A pergunta disparadora da entrevista foi:

-Como é para você ter uma ferida complexa?

No decorrer da entrevista, foram feitas perguntas acessórias, baseadas em um roteiro semiestruturado, com as seguintes questões:

-Há quanto tempo tem a ferida?
-Sempre foi cuidado na USF?
-Como seus familiares/amigos se portam em relação a você e sua ferida?
-Como é a questão do trabalho para você? Consegue trabalhar?
-E a vida social? Como se dá?

O critério de saturação teórica dos dados foi adotado, e ele foi discutido para cada categoria pelos pares pesquisadores do GEPEQ para a determinação do número de indivíduos entrevistados.

Todos os dados desta pesquisa estão depositados na *Open Science Framework* (OSF), disponível através do *link* <https://osf.io/5gxad/> e DOI 10.17605/OSF.IO/5GXAD. As identificações dos sujeitos foram feitas por códigos, a fim de manter o sigilo de sua identidade

### Análise dos dados

Os dados foram analisados pela técnica de análise de conteúdo clínico-qualitativa, por pares pesquisadores em reuniões de pesquisa do GEPEQ, obedecendo aos *Seven Steps* ^(12)^:

1. Material de edição – todas as entrevistas transcritas;
2. Leitura flutuante – o pesquisador faz uma leitura livre das experiências de vida relatadas;
3. Construção das unidades de análise – os pesquisadores identificam os significados, selecionam os fragmentos de fala e desenvolvem reflexões iniciais de cada fragmento;
4. Construção de códigos de sentido – agrupamento das unidades de análise semelhantes, estruturando, assim, os primeiros códigos de sentido;
5. Construção de categorias – organização do material para análise de todos os participantes com vistas ao agrupamento dos códigos de sentido;
6. Discussão – um diálogo com a literatura disponível;
7. Validade – reflexão crítica sobre os processos realizados em cada etapa.

## RESULTADOS

Foram incluídos e entrevistados oito usuários. Não houve desistência. As idades variaram entre 50 e 80 anos, (média de 65 anos), sendo dois do sexo masculino e seis do sexo feminino. Três usuários já passaram por amputação anterior; quatro vivem com diabetes (dos quais dois já passaram por amputações de membros); e os demais apresentam outras patologias vasculares. O período de duração das feridas variou de um a 50 anos, sendo que três pessoas possuíam feridas com um ano de duração, e os demais, mais de dez anos. Entre os entrevistados, três possuíam antecedente de amputação em membro, sendo dois em dedos dos pés, e um, em ambas as pernas. Somente uma pessoa tinha atividade laboral como auxiliar de serviços gerais. Em relação ao local de residência, quatro entrevistados necessitaram sair de suas residências por um período ou definitivamente, a fim de residir com outra pessoa devido à dependência total ou parcial para o autocuidado. Dois entrevistados demonstraram maior fragilidade e dificuldade emocional ao lidar com a ferida e outras questões pessoais. Nesses casos, a entrevista foi interrompida e os entrevistados foram encaminhados para avaliação psicológica na rede municipal.

Três categorias foram consolidadas, que seguem descritas e ilustradas com os relatos dos sentidos, significados e percepções das pessoas que convivem com a condição em relação à persistência de suas feridas:

1. Não tem mais vida social: sofrimento para além da dor física;
2. Comunicação paciente-profissional;
3. Religiosidade como estratégia de enfrentamento.

### 1) Não tem mais vida social: sofrimento para além da dor física

Além da dor física presente e permanente que acompanha os entrevistados, há o agravamento do sofrimento percebido, que se manifestou subjetivamente por sentimentos de perdas variadas (de autonomia, de vida social, do papel na família, *etc.*), por frustrações e por estigma.

[…] *é que a gente quer sair, voltar pra vida normal da gente, né?!* (P2)

[…] *minha vida, né, não tem mais vida social!* […] *acho que tudo isso mexe, sim* […] *nem tem onde ir sem calçado, né? Já há muito tempo não saio mais* […] *a gente se sente um pouco inútil, né?!* (P4)

*Eu me sinto muito ruim. Dá uma má sensação, você não fica à vontade. A aparência é feia. Ah, é como se alguma coisa em você está indo embora? Você quer esconder! É uma marca que marca mesmo! Daí você vai escondendo* […]. (P8)

A falta de perspectivas permeia a rotina das pessoas e traz desânimo e pessimismo.

[…] *eu tenho tristeza, desânimo, isso dói, queima, arde, coça. Dói dia e noite, tomar remédio não adianta, aí eu fico desanimado* […] *tomar remédio e tudo e não vê resultado.* (P7)

[…] *eu ficava* […] *sei lá, angustiada, desacorçoada, porque ela doía muito, né? Era meia funda. E queimava tanto a perna assim e pegava fogo na perna. E eu olhava assim, fazia de tudo e ela não melhorava.* (P1)

*Eu sofri muito, muita dor!* […] *quando eu vou lá no postinho [USF], que eu vou fazer o curativo do pé, eu fico nervosa, fico nervosa, pensando o pior. Eu não vou aguentar outra cirurgia, vai ser difícil eu fazer. Meu medo é isso.* (P5)

Há distorções entre o que se fala e o que se entende, e o impacto que isso traz para a pessoa emocionalmente envolvida pode acompanhá-la durante todo o período que ela permanece com a ferida. Disso emergiu uma categoria relacionada à comunicação:

### 2) Comunicação paciente-profissional

*Aí, antes de amputar o segundo dedo, o dr. explicou pra mim: “Você tem risco enorme de perder esse pé”. Ah, meu Deus, fiquei mais angustiado, né!* (P2)

[…] *teve uma médica que falou assim pra mim: “O que que adiantou você ficar com o pé e o pé não te servir pra nada?”. Porque ela disse assim: “Se você está com o pé, o médico não amputou seu pé, mas você não vai poder usar ele”.* (P4)

*E o médico falou pra mim que é do sangue, né, circulação.* (P3)

Parece existir uma dissonância entre o falar e o ouvir. O que é dito muitas vezes se distancia do que é ouvido e interpretado.

A crença da ferida incurável pode ser reforçada pela comunicação, como nas falas abaixo de P7 e de P6:

*O médico mesmo já falou que é crônico, não tem cura. Dá uma melhorada, aí depois volta* […] *crônico, porque já tem 50 anos* […]. (P7)

[O médico disse] *“Ah, a senhora pensa que a senhora vai ter cura? A senhora não vai ter cura, não* […]*”.* (P6)

Esta falta de perspectiva de cura abre portas tanto para a frustração quanto para fantasias e para a crença naquilo que é intangível. Isso se reflete nas falas sobre religião, das quais emergiu uma categoria relacionada com a religiosidade:

### 3) Religiosidade como estratégia de enfrentamento

A religião e a fé sempre estão presentes no cotidiano das pessoas, e isso pode impactar negativamente ou positivamente a vida dos indivíduos em tratamento das feridas complexas. A fé, nestes casos, tem duas vertentes: a fé que sustenta o otimismo e a espera pela cura; e a fé tendendo ao negativismo, que torna a pessoa vítima de castigo e punição.

*Não sei se você acredita ou não* […] *eu acho que fizeram alguma coisa pra mim.* (P1)

*Olha, eu sou evangélica, tenho muita fé em Deus* […]. *Entrego nas mãos de Deus, e espero que ele me abençoe.* (P5)

*Então, eu entrego pra Deus, Deus sabe o que faz. Então, uma hora ou outra vai sarar. Eu tenho fé.* (P1)

*Mas eu fiquei mais assim* […] *com aquela fé que eu estou sarando.* (P2)

[…] *quem me ajudou foi Deus. Deus é tudo, né?! Então, por Deus, pode ser que eu me cure, mas por mim, não.* (P6)

Há superstições correntes entre pacientes de feridas complexas de que esses agravos podem ser “castigos”, “sujeiras do sangue”, de que não existe tratamento ou cura, ou, ainda, sobre a iminência da morte em razão da cicatrização, como no caso da P1, que está com a ferida há mais de 30 anos e conta a história que segue, revelando medo da proximidade da morte.

*Eu não acreditava em mais nada!* […] *não sei se você acredita em benzedor, essas coisas* […] *que daí foi uma mulher [se] benzer lá, fechou a ferida [dela], só que ela não durou muito. A ferida fechou, mas a pessoa não durou muito.* (P1)

*Eu não sei se você acredita ou não, mas coisa assim, malfeita, eu acredito nesses negócios aí* […] *eu acho que fizeram alguma coisa pra mim.* (P1)

## DISCUSSÃO

Os relatos demonstraram desencontros na comunicação entre profissionais e pacientes. A partir da patologia das feridas, o indivíduo tem que alterar seus padrões e estilo de vida, e passa a viver em função de seu problema, renunciando às coisas que mais gostava. Ao refletirem sobre a necessidade de mudanças na rotina e no cotidiano, as falas dos entrevistados trazem à tona um dado clínico importante que explica uma das dificuldades no tratamento de feridas que é a adesão ao tratamento.

Durante as entrevistas, percebeu-se que a fé traz consequências ambíguas, tanto de otimismo quanto de pessimismo, e esses sentimentos estão condizentes com a evolução do quadro dessas pessoas.

Compreender a dimensão subjetiva do sofrimento de um paciente que convive com uma ferida complexa requer extrapolar as dimensões física, técnica e tecnológica do cuidado a esse agravo. Portanto, isso significa explorar elementos que fogem ao paradigma biomédico, estruturante da formação em saúde.

As dimensões social e subjetiva do cuidado à saúde são dele indissociáveis, e isso é um fato. Afinal, a doença “habita” um corpo, que está inserido em uma sociedade e nos comunica algo através da sua patologia, do seu adoecimento e da sua realidade social. Por isso, somente medicalizar é insuficiente. É preciso olhar para o que a doença e a saúde representam, quais os sentidos e significados experimentados e enfrentados, o que dizem do espaço vivido, da dinâmica social e individual^(13,14)^.

A investigação sobre as vivências dos pacientes com feridas complexas fez emergir a dor que vem da subjetividade, do que é dito nas entrelinhas da conversa. A dor, considerada o quinto sinal vital, não é somente física, mas persiste e se manifesta de diferentes formas, expondo a limitação imposta, a dependência adquirida e, de forma sutil, a expectativa e a desesperança que acompanham o usuário. Assim, surgiu a primeira categoria temática deste estudo:

### Não tem mais vida social: sofrimento para além da dor física

Conviver com o doente com ferida complexa e perceber seu sofrimento físico e psíquico nos faz refletir que esta condição traz uma série de mudanças na vida, não apenas da pessoa com uma ferida, mas também de seus familiares que, muitas vezes, não estão preparados para compreender todos os aspectos que envolvem este problema^(3)^.

Conduzir a vida levando consigo uma marca que necessita ser cuidada rotineiramente provoca efeitos angustiantes e muitas vezes desesperadores^(3)^, pois a marca corporal física que a ferida traz representa para o indivíduo um problema que não só é perceptível para ele, mas para todas as pessoas que o cercam, sendo fator limitante para as relações interpessoais^(15,16)^. A visão que a pessoa tem de si não se relaciona apenas à percepção e sentidos, mas envolve também figurações e representações mentais que o indivíduo possui de si e dos outros, do contato com ações e emoções advindas dessas experiências. Os indivíduos que possuem uma ferida complexa trazem o peso de uma doença visível que está à mostra na pele e uma tristeza que pesa a alma, causando um sofrimento psíquico e a perda da qualidade de vida^(3)^. Portanto, é uma dor subjetiva.

Estudos sobre as percepções de pacientes com feridas complexas apontam que a observação mais abrangente é a sobrecarga que os pacientes sofrem com suas feridas e como isso os leva a se tornarem observadores passivos de seus cuidados^(17)^.

No cotidiano das pessoas com feridas, há sofrimentos devido às dúvidas e angústias em relação ao tratamento, além de ansiedade em ver a evolução da ferida para uma melhora. Percebe-se que a ferida pode não ser apenas uma lesão física, mas algo que dói sem necessariamente precisar de estímulos sensoriais, uma marca, uma perda irreparável, ou seja, algo além de uma doença incurável^(3)^. Nesse sentido, os resultados de estudo realizado com pacientes que sofrem com lesões de membros inferiores corroboram os achados do presente trabalho, na medida em que seus participantes relatam que a preocupação surge da incerteza sobre a recuperação e o medo de complicações, levando à preocupação constante. Da mesma forma, a frustração decorre das limitações físicas e da lentidão no processo de cura, o que gera insatisfação e agrava o sofrimento emocional^(18)^.

O atendimento em saúde desse paciente é, via de regra, a oportunidade para acessar o cuidado que possa dirimir essas angústias. Entretanto, o que se observou foi que importantes dissonâncias podem ocorrer nesse encontro paciente-profissional, as quais podem não somente aprofundar as angústias já existentes, mas ainda causar novos complicadores. Essa percepção dos pacientes entrevistados fez surgir uma categoria relacionada à comunicação paciente-profissional.

### Comunicação paciente-profissional

O modo como se dá a comunicação de uma má notícia influencia diretamente as próximas etapas do tratamento, isto é, o seguimento do tratamento por parte do usuário é fortemente definido a partir da eficácia da comunicação^(19)^. Além dessas informações e de como são interpretadas, é preciso considerar a bagagem que pesa sobre a pessoa com ferida complexa, que pode trazer marcas profundas que acompanham a pessoa por um longo período, complicando o quadro, bem como contribuindo para os sentimentos negativos que permeiam o dia a dia dessas pessoas^(20,21)^.

A maneira como o profissional expressa a complexidade do caso pode levar o paciente a não acreditar nos processos terapêuticos necessários ao seu problema^(20)^.

Profissionais de saúde deveriam se familiarizar mais com tecnologias leves e relacionais, já que o ato de cuidar está intrinsecamente ligado à comunicação, que é uma tecnologia leve. Mais do que passar a informação correta, a comunicação terapêutica deve levar em conta a angústia, o prognóstico e a possibilidade de entendimento do usuário, percebidos através da escuta qualificada e dos sinais fornecidos através da subjetividade. O portador de feridas, que já vem com receio da cronicidade, pode muitas vezes distorcer a informação recebida, e essas informações podem se somar às crenças da ferida incurável.

As demandas subjetivas dos pacientes exigem dos profissionais atitudes psicodinâmicas, favorecendo o entendimento e acolhimento dessas demandas. Essas atitudes são também subjetivas, e são tão importantes quanto a própria postura protocolar de conduta clínica^(21)^.

Na ausência dessas atitudes profissionais, o que se pode identificar é que o sofrimento dos pacientes dá lugar a crendices com potencial de fazê-los desistirem do tratamento, mas, por outro lado, geram a necessidade de crença no sobrenatural para buscarem esperança e cura. Assim, a religiosidade foi uma categoria emergente das falas, demonstrando sua importância no processo terapêutico.

### Religiosidade como estratégia de enfrentamento

A crença religiosa é uma parte importante da cultura, dos princípios e dos valores utilizados pelas pessoas para dar forma a julgamentos e ao processamento de informações. A espiritualidade traz esperança e perspectivas de um futuro melhor, de viver com mais qualidade de vida e alimentando a esperança de cura^((22,23)^.

A fé e a religiosidade são temas recorrentes nos estudos que exploram as percepções de pessoas sobre o significado das feridas complexas nas suas vidas. Corroborando os achados desta pesquisa, artigo verificou que a espiritualidade foi exaltada como algo sagrado^(24)^. Os pacientes definiram Deus como ser acima de tudo, e a fé foi apontada como caminho para o bem-estar, que transcende a alma e os faz ter esperança na cura e na cicatrização da lesão considerada complexa. Entretanto, existem crendices a respeito das feridas complexas que funcionam de forma contrária, trazendo desesperança, posto que colocam a ferida como um “ajuste de contas moral” de Deus com o indivíduo.

Ao respeitarmos a importância prática que a espiritualidade, a religiosidade e a fé possuem do ponto de vista terapêutico, se queremos ir fundo na compreensão dos significados atribuídos por esses pacientes à sua doença, necessitamos compreender esse fenômeno do ponto de vista psicodinâmico. É comum, entre pessoas que passam por grandes desafios, sofrimentos e angústias, o uso de mecanismos de defesa do ego como a regressão, que funciona como a confiança da criança na proteção dos pais. É uma forma de se proteger do sofrimento e da negatividade que permeiam em casos complexos. A regressão é mecanismo de defesa caracterizado por um retorno do indivíduo às formas anteriores de desenvolvimento do pensamento e do estilo de relação com seu ambiente. Caracteriza-se por diminuição da capacidade para suportar frustrações, dependência do grupo social e, como nos casos dos entrevistados, pensamento mágico, no qual o paciente acredita e se entrega à onipotência do divino^(25,26)^.

Quando a equipe de saúde tem conhecimento psicodinâmico básico desses fenômenos e seus impactos no tratamento, pode melhor apoiar o indivíduo nos momentos mais desafiadores, como nos casos de frustração de suas expectativas, ou quando novas privações e limitações forem impostas. Será frustrante ao paciente que empenha esforços na fé reconhecer a frustração dos elementos mágicos outrora norteadores. Nesse momento, a equipe precisa estar atenta para acolher e buscar reavivar esperanças no paciente. A atitude psicoterapêutica denota ter uma postura de acolhimento às angústias do paciente. Importante ressaltar que a atitude psicoterapêutica dos profissionais de saúde não é fazer terapia, mas é ter uma postura que acolhe as angústias do paciente. É promover uma relação clínica, terapêutica e interpessoal, não protocolada, fundamental para o seguimento terapêutico técnico posterior^(22)^.

Por outro lado, ao falar livremente sobre os sentidos e significados da ferida na sua vida, esses pacientes puderam elaborar seus sofrimentos, ainda que momentaneamente, o que, certamente, por si só, já é um momento terapêutico.

### Limitações do estudo

As entrevistas deste estudo, por terem sido feitas por uma pesquisadora, que é enfermeira, na mesma rede assistencial onde os entrevistados são atendidos, podem carregar certo viés de resposta desses indivíduos, que, eventualmente, podem ter se omitido em alguma questão em face de constrangimento. Nesse sentido, tomou-se a precaução de que a entrevistadora não entrevistasse pacientes por ela atendidos, a fim de atenuar este eventual viés. Além desse cuidado metodológico, é necessário também considerar que se, por um lado, há esse risco de viés, por outro, a experiência da pesquisadora no assunto também lhe permite maior grau de aprofundamento na coleta de dados, devido à sua familiaridade com o tema e com o curso natural deste agravo. Assim, há que se atentar que, apesar da riqueza de resultados obtidos, pode haver ainda mais elementos a serem explorados, para o que se sugere a continuidade desta pesquisa através de entrevistador(a) não relacionado(a) ao serviço.

### Contribuições para a área da saúde, enfermagem ou políticas públicas

Os resultados deste estudo podem oferecer aos profissionais de saúde elementos subjetivos coletados das vivências de pessoas que convivem com feridas complexas, e isso pode nortear o cuidado a esses pacientes de modo mais coerente com suas necessidades. Essa singularidade, além de tornar o encontro paciente-profissional mais acolhedor, permitirá ao profissional de saúde transformar o cuidado meramente biologicista (focado na ferida) em um ato que considere a integralidade dessas pessoas. Atentar para a subjetividade das pessoas que possuem feridas, portanto, é o caminho para o reconhecimento de seu sofrimento e respeito às suas inúmeras perdas e limitações.

Em relação, especificamente, à formação de profissionais da APS, o presente estudo contribui no sentido de apontar caminhos para o cuidado centrado no paciente. Sabe-se que, apesar dos inúmeros avanços tecnológicos e de conhecimento no cuidado às feridas, alguns fatores são desafiadores para o seu enfrentamento e contribuem para o agravamento, como uso de tabaco, apresentar restrição alimentar, sinais de infecção, características do odor e avaliação da dor^(27)^. A construção e o fortalecimento do vínculo paciente-profissional parecem ser cruciais na medida em que isso é central para adesão do usuário ao serviço e às recomendações profissionais. Entretanto, há que se considerar que as dificuldades e mudanças no cotidiano da pessoa que convive com uma ferida complexa por período prolongado, bem como as repercussões disso para o indivíduo e família, não são nada facilitadores dessa adesão; pelo contrário. A desesperança que permeia os dias, os procedimentos sem previsão de término e a frustração com os cuidados sem progresso fazem parte do cotidiano dessas pessoas durante meses e anos.

Quando o profissional de saúde tem consciência disso, certamente se solidariza, inclui no plano de tratamento meios para contornar dificuldades práticas que extrapolam o cuidado meramente técnico e acolhe esse usuário de forma diferenciada, centrando seu cuidado em cada paciente de forma singular. Os relatos deste estudo contribuem, portanto, apontando alguns desses significados que a ferida possui para quem a carrega e, dessa forma, prepara o profissional da APS para seu enfrentamento. Do ponto de vista organizacional, as unidades de APS, reconhecendo os significados desafiadores ora relatados no presente estudo, que o convívio com as feridas traz a quem as carrega, podem se organizar para receber esses pacientes de forma ágil e com a maior privacidade possível nas dependências da unidade, evitando longas esperas em salas coletivas. Além disso, podem fazer visitas domiciliares com a periodicidade adequada a cada caso e, sobretudo, promover espaços de discussão sobre o cuidado centrado no paciente para seus trabalhadores.

## CONSIDERAÇÕES FINAIS

A ferida complexa, persistente por período prolongado, além da marca na pele, provoca uma tristeza que pesa a alma, trazendo sofrimento psíquico consequente das perdas e adaptações que precisa fazer para se adequar à sua realidade.

O conhecimento dos significados que essas feridas têm para os pacientes por ela acometidos é essencial para que os profissionais envolvidos adotem atitude psicoterapêutica nesses tratamentos, o que tende a aumentar a resolutividade dos casos, já que seu manejo vai além do conhecimento fisiopatológico.

Os sofrimentos vividos pelas pessoas com feridas complexas devem ser reconhecidos pelos cuidadores desses pacientes, a fim de qualificar a relação terapêutica na medida em que, ao considerá-los, o profissional pode aproximar suas recomendações da realidade do paciente, solidarizar-se com suas vivências e, ainda, aperfeiçoar a comunicação paciente-profissional, imprimindo ao seu discurso coerência com a subjetividade de cada caso.

## Data Availability

Os dados de pesquisa estão disponíveis em repositório: https://doi.org/10.17605/OSF.IO/5GXAD.
